# The impact of socioeconomic factors on pancreatic cancer care utilization

**DOI:** 10.1371/journal.pone.0320518

**Published:** 2025-05-07

**Authors:** Masoud Khani, Mohammad Assadi Shalmani, Amirsajjad Taleban, Susan Tsai, Mochamad Nataliansyah, Mohammed Aldakkak, Jake Luo

**Affiliations:** 1 Health Informatics Program, Zilber School of Public Health, University of Wisconsin-Milwaukee, Milwaukee, Wisconsin, United State of America; 2 Division of Surgical Oncology, Department of Surgery, Ohio State University Comprehensive Cancer Center, Columbus, Ohio, United State of America; 3 Department of Surgery, Medical College of Wisconsin, Milwaukee, Wisconsin, United State of America; Georgetown University, UNITED STATES OF AMERICA

## Abstract

**Background:**

Pancreatic cancer carries a dismal prognosis, with socioeconomic factors significantly impacting patient outcomes. This study investigates the influence of socioeconomic determinants on access to specialized pancreatic cancer care and utilization rates in southeast Wisconsin.

**Methods:**

We analyzed a dataset of 5,847 pancreatic cancer patients from the Froedtert & the Medical College of Wisconsin health system (2000-2023). Patient demographics were compared to the broader health system population. Utilization of specialized pancreatic cancer care was calculated for each patient’s zip code of residence. Linear and multivariate regression analyses were conducted to assess the association between socioeconomic factors (white population, income, education, insurance, Area Deprivation Index) and zip code-level utilization rates.

**Results:**

Pancreatic cancer patients were older (mean age 66.3 vs. 46.5 years), predominantly male (52.7%), and disproportionately White (83.2% vs 63.6%) compared to the general population. Notably, patients residing in zip codes with the lowest median household income (<$42,000) had a 0.15% utilization rate, while those in the wealthiest areas (>$87,000) showed a 0.14% rate. Interestingly, utilization dipped to its lowest point (0.068%) in areas with median incomes between $53,100-$59,300. Initial analysis suggested that higher education levels, private insurance, and higher median incomes were linked to increased utilization. However, after accounting for other factors, only the Area Deprivation Index (ADI) and the percentage of the White population remained significant predictors. Specifically, a one-unit increase in ADI (indicating greater neighborhood disadvantage) was associated with a 0.0015% decrease in specialized care utilization (p < 0.05). Similarly, a 1% increase in the White population within a zip code was linked to a 0.0014% reduction in utilization (p < 0.05).

**Conclusion:**

Our findings reveal that neighborhood-level socioeconomic disadvantage, as captured by the ADI, is a strong independent predictor of reduced access to specialized pancreatic cancer care in southeast Wisconsin. Furthermore, factors such as education level, income, and insurance status are significantly associated with increased utilization of these vital services. These results underscore the urgent need for targeted interventions to address these inequities and ensure that all pancreatic cancer patients have equal access to potentially life-saving care, regardless of their socioeconomic background.

## 1. Introduction

Pancreatic cancer is an aggressive malignancy with poor overall survival [1,2]. In the United States, pancreatic cancer represents approximately 3.3% of all cancer diagnoses but accounts for a disproportionate 8.5% of cancer-related deaths, underscoring its significant public health burden [3]. The 5-year overall survival rate for pancreatic cancer remains dismal at approximately 13%, showing only modest improvement from 7% over the past decade despite significant advances in cancer management [3].

This stark contrast highlights the urgent need for enhanced research efforts and novel treatment strategies to combat this devastating disease, which is currently the 3rd leading cause of cancer-related death in the United States and is expected to become the 2nd by 2030 [4,5].

Social determinants of health (SDOH) have been extensively documented as fundamental drivers of health outcomes across various diseases [6–8]. These determinants encompass socioeconomic status, education levels, geographic location, and access to healthcare resources, all of which significantly influence health outcomes [9,10]. In the context of pancreatic cancer, previous investigations have identified substantial disparities in hospital utilization, treatment access, and patient outcomes correlated with these social determinants [11,12]. The aggressive nature of pancreatic cancer, combined with challenges in early detection and limited therapeutic options, amplifies the impact of these social determinants on patient outcomes. Evidence indicates that socioeconomic status, including income, education, and geographic location, is crucial in determining access to essential services, including early detection tools, specialized surgical interventions, and advanced treatment modalities [12–14].

While robust evidence establishes the correlation between socioeconomic factors and healthcare outcomes [10,15], the specific mechanisms through which these determinants affect access to specialized oncological services and subsequent patient outcomes can vary significantly by geographic region [7,16,17]. This regional variation in SDOH impact necessitates localized investigation to develop targeted interventions effectively. Notably, despite the documented importance of understanding regional SDOH patterns, there exists a critical knowledge gap regarding their specific influence on pancreatic cancer outcomes in southeast Wisconsin.

Our investigation addresses this gap by examining how socioeconomic disparities influence access to specialized oncological services and treatment outcomes in southeast Wisconsin’s pancreatic cancer patient population. We hypothesize that socioeconomic factors significantly impact the utilization of specialized oncological services, resulting in measurable disparities in treatment outcomes and survival rates. Specifically, we predict that patients from lower socioeconomic backgrounds experience reduced access to advanced diagnostic and therapeutic modalities, with implications for their clinical outcomes.

## 2. Methodology

### 2.1. Ethics statement

We analyze data from the Clinical Research Data Warehouse (CRDW) at the Clinical & Translational Science Institute (CTSI). No human or animal subjects were involved. This study received ethical approval from the Institutional Review Board of the Medical College of Wisconsin/Froedtert Hospital (protocol number PRO00039895). To safeguard patient privacy and data integrity, all patient data was managed securely through the Clinical Research Data Warehouse, a component of Southeast Wisconsin’s Clinical and Translational Science Institute of Southeast Wisconsin (UL1TR001436). This system provides a secure, continuously updated mirror of the complete electronic health record system housed within a Jupyter environment.

All patient information was de-identified to ensure confidentiality. Dates within the dataset were systematically shifted to prevent the identification of individual patients, and each patient was assigned a unique patient hash for anonymity. The data were accessed for research purposes on January 06, 2024. The authors did not have access to any information that could identify individual participants during or after data collection.

### 2.2. Data source

The Froedtert & the Medical College of Wisconsin health system, which serves over 1.9 million unique patients in southeast Wisconsin, provided the primary dataset. Adult patients diagnosed with pancreatic cancer between 2000 and 2023 were identified using International Classification of Diseases (ICD) codes: Ninth Revision (157.X) and Tenth Revision (C25.0–C25.9). Specialized pancreatic cancer care refers to care provided within the oncology specialty, including diagnosis, treatment planning, and management by oncology specialists.

The dataset included demographic information such as diagnosis date, age, gender, race, ethnicity, zip code of residence, and insurance status at the closest encounter date. Comparative demographic data for all other patients within the health system who did not have pancreatic cancer were also collected to establish a baseline representation of the general patient population.

Regional socioeconomic and demographic data from 2016 to 2021 were obtained from the U.S. Census Bureau [18]. This external data provided zip code-level insights, enabling comparisons between patients receiving oncology services and the broader population.

### 2.3. Social determinants of health variables

The selection of specific SDOH variables was guided by established frameworks in health disparities research and prior evidence demonstrating their significant association with cancer outcomes. The chosen variables encompass five critical domains of social determinants:

1. **College education rate:** This variable serves as a key indicator of educational attainment, which is closely associated with health literacy and the ability to navigate healthcare systems effectively. Higher education levels enable individuals to better understand medical information, make informed health decisions, and engage proactively with specialized care services. Research has demonstrated a strong link between education and improved health outcomes across various cancer types, including pancreatic cancer [19].
2. **Area Deprivation Index (ADI)** [20]: The ADI provides a composite measure of neighborhood-level socioeconomic disadvantage, incorporating factors such as income, education, employment, and housing quality. It captures the cumulative effects of multiple social disadvantages on health outcomes. Unlike individual SDOH variables, ADI reflects persistent structural inequities, offering temporal stability and facilitating inter-regional comparisons. Its standardized nature and validated psychometric properties make it a robust tool for assessing the multiplicative and non-linear effects of co-occurring social disadvantages. Incorporating ADI alongside other SDOH variables enhances the analytical framework, providing a comprehensive perspective on the interplay of socioeconomic factors in shaping access to care.
3. **White population (%):** The proportion of White residents was included to examine racial disparities in access to specialized pancreatic cancer care. This variable reflects the role of racial demographics in cancer outcomes, healthcare access, and participation in clinical trials. Extensive literature highlights systemic inequities and structural barriers faced by racial minorities, making this variable essential for understanding disparities within the population served by the healthcare system [21].
4. **Median household income ($):** Median income was selected as a direct measure of economic access to healthcare services. It represents financial resources available within a community, which can significantly influence access to specialized oncology care, treatment options, and adherence to therapeutic regimens. Higher income levels are often associated with fewer delays in care initiation, greater access to advanced treatments, and improved survival outcomes [22].
5. **Privately insured (%):** Insurance status is a critical determinant of healthcare access, particularly in specialized oncology care where the availability and timing of treatment options are often dictated by insurance coverage. The percentage of privately insured individuals within a zip code was included as a measure of financial and systemic access to healthcare resources, reflecting disparities in the ability to obtain timely and comprehensive cancer care [23].

These variables were selected over other potential SDOH indicators based on: (a) their demonstrated predictive value in cancer outcomes, [24] (b) the availability of reliable, standardized data at the zip code level, and (c) their potential modifiability through targeted interventions [25]. Census-derived population data enabled robust comparisons between patients utilizing oncology services and the broader health system population.

### 2.4. Analytical framework

Zip code-level data were categorized into income brackets as defined by DATAUSA, [26] facilitating structured comparisons across socioeconomic strata. By integrating Froedtert Health System data with U.S. Census Bureau data, this study combined patient-level and neighborhood-level factors to provide a comprehensive analysis of pancreatic cancer care utilization.

All pancreatic cancer patients who visited the hospital were considered as utilizing specialist pancreatic cancer care, as these services involve advanced, multidisciplinary expertise tailored to the diagnosis and treatment of this complex disease.

Specialist pancreatic cancer care utilization was defined as the use of advanced, multidisciplinary services tailored to the diagnosis and treatment of this complex disease. Utilization rates were calculated at two levels:

1. **Population level:** The number of pancreatic cancer patients receiving care within the health system divided by the total population of the corresponding zip code, based on U.S. Census data.
2. **Hospital level:** The number of pancreatic cancer patients divided by the total number of hospital patients from each zip code, reflecting access and utilization specific to the hospital system.

This dual-level approach allowed us to explore community-wide trends and hospital-specific dynamics in pancreatic cancer care access.

### 2.5. Statistical analysis

Descriptive statistics were used to characterize the study population and compare utilization rates across socioeconomic groups. First, we used chi-square tests and odds ratios to examine differences in the demographic characteristics between pancreatic cancer patients and the general health system population. For continuous variables such as age, independent t-tests were performed.

To examine the relationship between socioeconomic factors and pancreatic cancer care utilization, zip code data categorized into income brackets were analyzed. Means and 95% confidence intervals were calculated for each socioeconomic indicator, and effect sizes were determined using Pearson correlation coefficients to assess the strength of associations.

Next, Linear regression models were employed to analyze the relationship between zip code-level socioeconomic indicators and utilization rates. Multivariate regression models were subsequently developed to analyze the combined effect of these socioeconomic factors on pancreatic cancer care utilization. Both unstandardized and standardized beta coefficients were calculated to quantify the relative impact of each socioeconomic factor.

All statistical analyses were conducted using Python 3.10. A p-value of 0.05 or less was considered statistically significant. Through this comprehensive analysis, we sought to elucidate the socioeconomic determinants that contribute to disparities in access to specialized pancreatic cancer services and, ultimately, patient outcomes within the southeast Wisconsin region.

## 3. Results

### 3.1. Patient demographics

The study population consisted of 5,847 adult patients diagnosed with pancreatic cancer between 2000 and 2023 within the Froedtert & the Medical College of Wisconsin health system. Table 1 provides the demographics of the study population, with a comparison to the overall patient population of the health system.

**Table 1:** Demographic and socioeconomic characteristics of patients in the health system compared to pancreatic cancer patients.

Variable	Froedtert Hospital	Pancreatic Cancer	OR	95% CI	P-Value
**Age**	46.50 (±22.73)	66.33 (±12.35)	–	–	<0.001
**Number of Patients**	1,967,242	5,847	–	–	–
**Sex**					
Female	1,038,806 (52.81)	2764 (47.27)	0.8	[0.76,0.84]	<0.001
Male	923,808 (46.96)	3083 (52.73)	1.26	[1.2,1.33]	<0.001
Unknown	4,628 (0.24)	0	0	[0.0, nan]	<0.001
**Race**					
White or Caucasian	1,250,265 (63.55)	4863 (83.17)	2.83	[2.65,3.04]	<0.001
Unknown	329,286 (16.74)	249 (4.26)	0.22	[0.19,0.25]	<0.001
Black or African American	253,856 (12.90)	534 (9.13)	0.68	[0.62,0.74]	<0.001
Other	73,057 (3.71)	106 (1.81)	0.48	[0.39,0.58]	<0.001
Asian	40,565 (2.06)	65 (1)	0.53	[0.42,0.68]	<0.001
Multiracial	7,421 (0.38)	11 (0.19)	0.5	[0.28,0.9]	0.0132
American Indian or Alaska Native	6,463 (0.33)	9 (0.15)	0.47	[0.24,0.9]	0.014
Patient Refused	4,819 (0.24)	9 (0.15)	0.63	[0.33,1.21]	0.175
Native Hawaiian or Other Pacific Islander	1,510 (0.08)	1 (0.02)	0.22	[0.03,1.58]	0.102
**Ethnicity**					
Non-Hispanic	1,534,084 (77.98)	5480 (93.72)	4.22	[3.79,4.69]	<0.001
NI	238,869 (12.14)	176 (3.01)	0.22	[0.19,0.26]	<0.001
Hispanic	97,581 (4.96)	122 (2.09)	0.41	[0.34,0.49]	<0.001
**Insurance Type**					
Private	846,112 (43.01)	2077 (35.52)	0.73	[0.69,0.77]	<0.001
Public	628,929 (31.97)	3579 (61.21)	3.36	[3.19,3.54]	<0.001
Unknown	417,820 (21.24)	74 (1.27)	0.05	[0.04,0.06]	<0.001
SELFPAY	52,460 (2.67)	40 (0.68)	0.25	[0.18,0.34]	<0.001
Other	21,921 (1.11)	77 (1.32)	1.18	[0.95,1.48]	0.17

Significant differences were observed in age and gender distribution between the pancreatic cancer cohort and the general population. Pancreatic cancer patients were significantly older, with a mean age of 66.33 years (±12.35) compared to 46.50 years (±22.73) in the general population. Furthermore, a higher proportion of pancreatic cancer patients were male (52.7%) compared to the general population (47.0%). This difference was statistically significant, with an OR of 1.26 (95% CI: 1.20-1.33; P < .001).

Racial demographics revealed notable disparities. The pancreatic cancer cohort had a disproportionately higher representation of White patients (83.2%) compared to the broader health system population (63.6%), resulting in an OR of 2.83 (95% CI: 2.65-3.04; P < .001). Conversely, patients with unknown racial backgrounds were significantly underrepresented in the pancreatic cancer group (4.3% vs. 16.7%; OR: 0.22, 95% CI: 0.19-0.25; P < .001). Similarly, Black patients (9.1% vs. 12.9%; OR: 0.68, 95% CI: 0.62-0.74; P < .001) and other racial groups were underrepresented in the pancreatic cancer cohort, indicating significant racial stratification.

Ethnicity analysis demonstrated that non-Hispanic individuals were more prevalent in the pancreatic cancer group (93.7%) compared to the general population (78.0%), corresponding to an OR of 4.22 (95% CI: 3.79-4.69; P < .001).

Insurance coverage also differed significantly between the two groups. Pancreatic cancer patients were more likely to have public insurance (61.2% vs. 32.0%; OR: 3.36, 95% CI: 3.19-3.54; P < .001) and less likely to have private insurance (35.5% vs. 43.0%; OR: 0.73, 95% CI: 0.69-0.77; P < .001).

These findings reveal distinct demographic and socioeconomic profiles among pancreatic cancer patients within our health system, highlighting potential disparities in disease incidence and access to care. This information is crucial for informing future research efforts, developing targeted prevention strategies, and tailoring care practices to address the specific needs of diverse pancreatic cancer populations.

### 3.2. Income level analysis

Our analysis of zip code-level income data revealed significant disparities in the utilization of specialized pancreatic cancer care. Table 2 provides a detailed breakdown of utilization rates alongside key socioeconomic factors, including college education levels, the percentage of white individuals, and the proportion of privately insured residents within each income bracket. By examining income alongside these additional variables, this analysis offers a multidimensional perspective on how socioeconomic and demographic factors collectively influence access to specialized pancreatic cancer care, shedding light on critical disparities within the healthcare system.

**Table 2:** Impact of Income on Oncology Clinic Utilization and Social Determinants.

	Income ^a^								
Categories	<42K	42K–53K	>53K–59K	>59K–67K	>67K,77K	>77K,87K	>87K	Effect size^b^	95% CI
Utilization	0.15	0.097	0.068	0.069	0.10	0.11	0.14	0.19	0.1–0.2
	(0.11–0.19)	(0.072–0.12)	(0.05–0.08)	(0.054–0.084)	(0.07–0.12)	(0.087–0.13)	(0.11–0.16)		
College Educated	6.86	9.94	10.73	11.87	12.61	14.8	20.02	0.64	18.8–21.3
	(5.0–9.0)	(9.0–11.0)	(10.0–12.0)	(11.0–13.0)	(12.0–14.0)	(13.0–16.0)	(19.0–21.0)		
White	39.9	83.22	87.78	90.59	91.14	92.92	91.55	0.379	90–93.1
	(21.0–58.0)	(77.0–90.0)	(84.0–91.0)	(88.0–93.0)	(89.0–93.0)	(91.0–95.0)	(90.0–93.0)		
Private Insurance	48.95	64.36	68.58	72.17	77.54	82.45	85.16	0.758	84.1–86.3
	(43.0–55.0)	(61.0–68.0)	(67.0–70.0)	(71.0–74.0)	(76.0–79.0)	(81.0–83.0)	(84.0–86.0)		

^a^Values are presented as percentages (95% CI).

^b^Effect sizes were calculated using Pearson correlations (r). The effect size is low if r is approximately 0.1, medium if is approximately 0.3, and large if > 0.5.

The lowest utilization rates were observed in the middle-income categories (>53K-59K and> 59K-67K), while both the lowest (<42K) and highest income groups (>87K) displayed higher utilization. Furthermore, the proportion of individuals with a college education, identifying as White, and possessing private insurance all increased with rising income levels.

**Fig 1:**
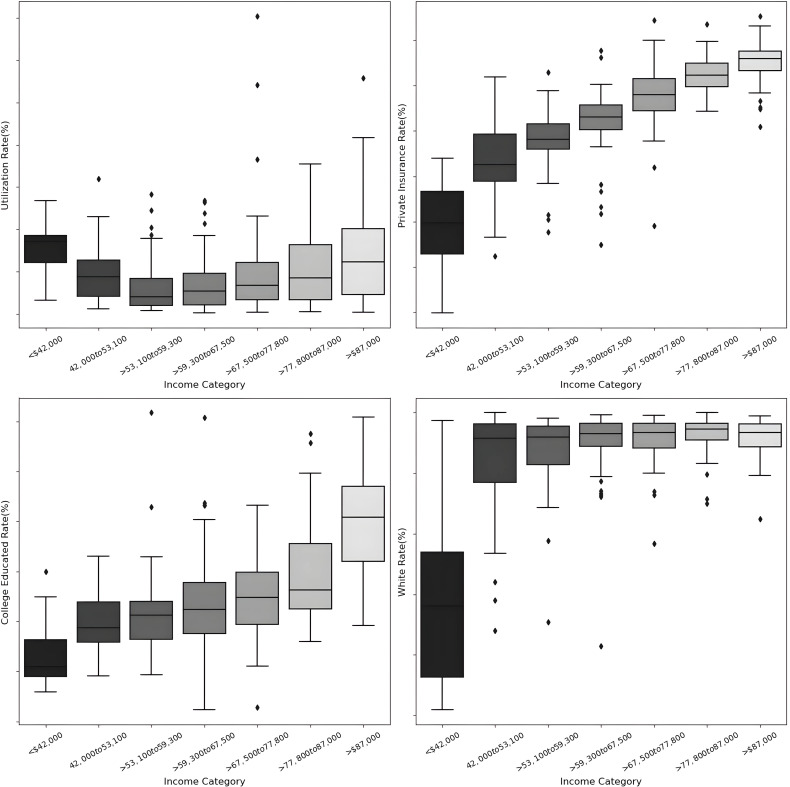
Correlation of Socioeconomic Indicators with Healthcare Utilization and Demographics: These box plots illustrate the relationships between different income categories and key socioeconomic indicators: the utilization rate of oncology services, the rate of private insurance coverage, the proportion of college-educated individuals, and the percentage of the White population. Each graph shows the median values, quartiles, and outliers, indicating the distribution and central tendency within each income bracket. The plots reveal that higher income categories generally correspond with increased utilization of oncology services, higher private insurance and college education rates, and a larger percentage of the White population.

Our analysis reveals a non-linear relationship between income and oncology service utilization. Utilization was lowest in the $53,100-$59,300 income bracket, while the highest income bracket (>$87,000) demonstrated significantly greater utilization (p <  0.001). This suggests that while income influences access, other factors may be at play. Increased utilization in higher income brackets may reflect greater healthcare-seeking behavior, better insurance coverage, or improved healthcare system navigation. Fig 1 highlights the distribution of pancreatic cancer care utilization rates across median household income brackets

To further investigate these socioeconomic determinants and their impact on utilization, we performed a linear regression analysis. This allowed us to quantify the influence of factors such as income, education, insurance status, and area deprivation on oncology service use.

### 3.3. Regression analysis of social determinants on clinic utilization

#### 3.3.1. Community-level prevalence.

Table 3 summarizes the results of the linear regression analysis examining the relationship between socioeconomic factors and pancreatic cancer utilization rate. The analysis revealed a significant relationship between socioeconomic indicators and oncology service utilization. The college education rate, median income, and percentage of privately insured individuals within a zip code were all positively associated with higher pancreatic cancer utilization rates.

**Table 3:** Regression Coefficients for Social Determinants of Community-Level Pancreatic Cancer Care Utilization.

Variable	Coefficient	Standard Error	Lower bound	Upper bound	P value
**College education rate**	0.0026	1.00** × **10^***−*** ***−*** 3^	1.00** × **10^ − ^−^3^	4.00** × **10^ − 3^	0.003
**ADI**	−0.0007	0.00E + 00	−1.00** × **10^ − 3^	0.00E + 00	<0.001
**White, %**	−0.0004	0.00E + 00	−1.00** × **10^ − 3^	0.00E + 00	0.169
**Median income, $**	1.01** × **10^ − 8^	2.67** × **10^ − 9^	4.90** × **10^ − 9^	1.54** × **10^ − 8^	<0.001
**Privately insured, %**	0.001	0.00E + 00	0.00E + 00	0.002	0.028

An analysis of the predictors of oncology service utilization for pancreatic cancer patients revealed several significant socioeconomic determinants. For every 1% increase in the rate of college education, the utilization rate increased by 0.26 percentage points (β =  0.0026, p <  0.05), after adjusting for other covariates. Moreover, the proportion of privately insured individuals emerged as a notable predictor, where a 1% rise in private insurance coverage corresponded to a 0.001 percentage point increase in service utilization (β =  0.001, p <  0.05).

Income also exhibited a significant positive association with oncology service use. Specifically, each $10,000 increment in median income was associated with a 1.01 ×  10^-8^ percentage point increase in utilization (β =  1.01 ×  10^-8^, p <  0.001). In contrast, living in deprived areas, as indicated by the Area Deprivation Index (ADI), showed a strong negative correlation with utilization rates. A 1-unit increase in ADI was linked to a 0.0007 percentage point decrease in utilization (β =  -0.0007, p <  0.001).

Interestingly, the proportion of White individuals in the population was not a statistically significant predictor of oncology service use when controlling for other socioeconomic variables. A 1 percentage point increase in the White population corresponded to a 0.0004 percentage point decrease in utilization, this relationship did not reach statistical significance.

**Fig 2:**
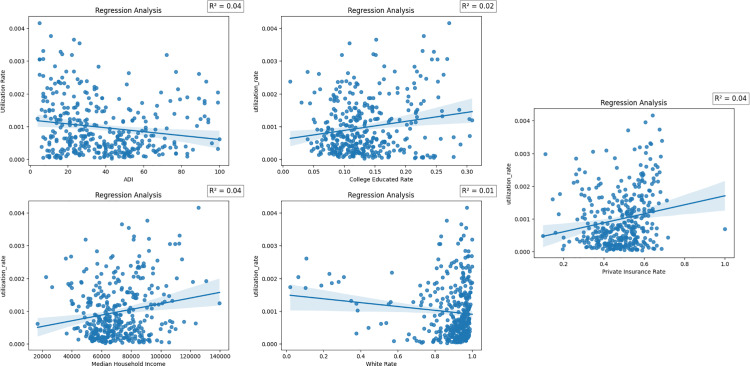
Regression analyses of community-level pancreatic cancer care utilization rates against key social determinants of health. Plots show relationships with Area Deprivation Index (ADI), college education rate, median household income, White population rate, and private insurance rate. Regression lines depict trends, and shaded areas represent 95% confidence intervals, with corresponding R² values indicating the proportion of variance explained by each predictor.

Overall, these results underscore the importance of socioeconomic factors, including educational attainment, insurance coverage, income, and neighborhood deprivation, in influencing access to specialized oncology services for pancreatic cancer care. Fig 2 provides a visual summary of the linear relationships between these key predictors and service utilization rates.

#### 3.3.2. Hospital-level utilization.

Table 4 focuses on hospital-level utilization, analyzing the subset of individuals who actively sought care within the studied health system and developed pancreatic cancer. This distinction highlights two complementary dimensions of healthcare access. Table 3 examines community-level patterns, reflecting the broader burden of pancreatic cancer care and capturing disparities in accessibility and utilization across socioeconomic and demographic groups within the general population. In contrast, Table 4 delves into the hospital-specific context, providing insights into the characteristics of patients who navigate the system for specialized care. It explores how socioeconomic factors, insurance coverage, and referral pathways shape access within the hospital’s catchment area, offering a more targeted perspective on disparities that influence both disease burden and treatment dynamics at the institutional level.

**Table 4:** Regression Coefficients for Social Determinants of Hospital-Level Pancreatic Cancer Care Utilization.

Variable	Coefficient	Standard Error	Lower bound	Upper bound	P value
**College education rate**	−0.029343	0.009004	−0.047051	−0.011636	0.0012
**ADI**	0.000120	0.000051	0.000020	0.00022	0.0186
**White, %**	0.020614	0.007818	0.005238	0.035989	0.0087
**Median income, $**	−1.212292** × **10^ − 07^	7.044365** × **10^ − 08^	−2.597607** × **10^ − 07^	1.730225** × **10^ − 08^	0.086
**Privately insured, %**	−0.029170	0.009710	−0.048264	−0.010075	0.002

A 1% increase in the rate of college education was associated with a significant 0.0293 percentage point decrease in utilization among hospital patients (β =  -0.0293, p <  0.01), suggesting that individuals from areas with higher educational attainment may seek care outside the studied system, possibly reflecting differences in referral patterns or access preferences. Similarly, a 1% rise in private insurance coverage was linked to a 0.0292 percentage point decrease in utilization (β =  -0.0292, p <  0.01), which might indicate that privately insured patients have access to a wider range of healthcare providers beyond the system studied.

Conversely, the proportion of White individuals exhibited a positive association, with a 1% increase correlating to a 0.0206 percentage point rise in utilization (β =  0.0206, p <  0.01). This finding may reflect demographic differences in care-seeking behavior or access patterns among White populations. Furthermore, the Area Deprivation Index (ADI) showed a small but significant positive association, where a 1-unit increase in ADI corresponded to a 0.00012 percentage point rise in utilization (β =  0.00012, p <  0.05), highlighting that individuals from more deprived areas are more likely to seek care within the system, potentially reflecting reliance on centralized or public healthcare resources.

Median income, while showing a negative trend (β =  -1.21 ×  10^-7^), did not reach statistical significance (p =  0.086). This suggests that, unlike the broader community-level trends, income may play a less direct role in determining care access within the hospital system.

**Fig 3:**
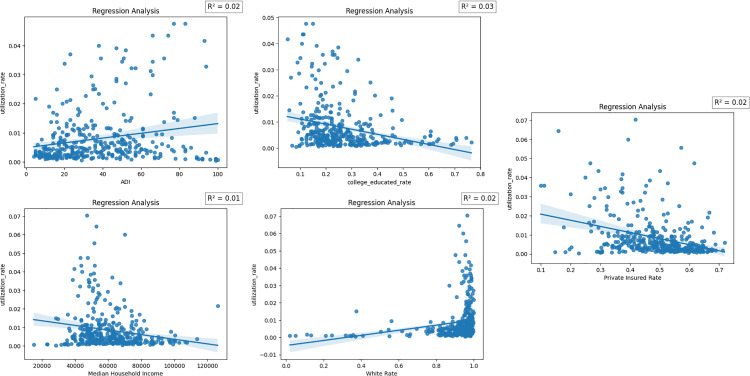
Regression analyses of hospital-level pancreatic cancer care utilization rates against key social determinants of health.

These results underscore the nuanced differences in the factors influencing hospital-level utilization compared to community-level prevalence, highlighting the complex interplay between socioeconomic status, demographic characteristics, and insurance coverage in shaping access to specialized oncology care. Together, Tables 3 and 4 offer complementary perspectives on pancreatic cancer care utilization, with Table 3 capturing community-wide trends and Table 4 providing a focused analysis of hospital-specific dynamics. Fig 3 presents a visual summary of the linear relationships between key predictors and hospital-level utilization rates, further illustrating these patterns.

### 3.4. Multivariable regression

Given the complex interplay of socioeconomic factors influencing pancreatic cancer care utilization, we developed multivariable models to better understand these relationships at both the community and hospital levels.

#### 3.4.1. Community-level utilization.

The findings revealed the ADI and the percentage of the white population as significant. However, other variables, such as the rates of college education, median income, and private insurance, did not exhibit a substantial independent correlation with service utilization. Table 5 provides a detailed analysis of these relationships, offering insights into the relative contributions of each socioeconomic factor.

**Table 5:** Multivariable Regression Coefficients for Social Determinants Impacting Utilization on population level.

Variable	Coefficient	Standard Error	Lower bound	Upper bound	P-value
**College education rate**	−0.0008	0.001	−0.003	0.002	0.494
**ADI**	−1.54** × **10^ − 05^	5.39** × **10^ − 06^	−2.6** × **10^ − 05^	−4.79** × **10^ − 06^	0.005
**White, %**	−0.0014	0.00	−0.002	−0.001	0.001
**Median income, $**	5.016** × **10^ − 09^	4.96** × **10^ − 09^	−4.75** × **10^ − 09^	1.48** × **10^ − 08^	0.313
**Privately insured, %**	−0.0011	0.001	−0.003	0.001	0.265

For each unit increase in ADI, utilization decreased by 1.54 x 10 ⁻ ⁵ percentage points (β =  -1.54 x 10 ⁻ ⁵, p <  0.05). Similarly, each 1% increase in the White population corresponded to a 0.0014 percentage point decrease in utilization (β =  -0.0014, p <  0.05). The ADI and white percentage both showed the negative influence of lower socioeconomic status and racial demographics on access to specialized oncology care on both linear and multivariate levels.

On the other hand, the analysis did not demonstrate a statistically significant independent effect of median income, education level, or insurance status on utilization after accounting for the influence of other predictors. A 1% increase in college education rate was associated with a 0.0008 percentage point *decrease* in utilization (β =  -0.0008, p =  0.494), while a similar increase in private insurance corresponded to a 0.0011 percentage point decrease (β =  -0.0011, p =  0.265). The effect of median income was even smaller and non-significant.

This suggests that while these factors may play a role in access to care, their impact is mediated through other socioeconomic determinants, such as neighborhood deprivation and racial demographics.

#### 3.4.2. Hospital-level utilization.

The multivariable regression analysis at the hospital level provided insights into the factors influencing access to pancreatic cancer care among individuals who actively sought care within the studied health system and subsequently developed pancreatic cancer. Unlike the community-level analysis, which reflects broader population-level trends and care-seeking behavior across the general population, the hospital-level analysis focused specifically on those who developed pancreatic cancer. It highlighted distinct patterns related to referral dynamics, care pathways, and systemic access within the hospital’s catchment area, offering a more granular perspective on the challenges faced by this patient cohort.

Table 6 presents the multivariable regression coefficients for hospital-level utilization, detailing the relative contributions of key social determinants in shaping access to specialized pancreatic cancer care.

**Table 6:** Multivariable Regression Coefficients for Social Determinants of Hospital-Level Pancreatic Cancer Care Utilization.

Variable	Coefficient	Standard Error	Lower bound	Upper bound	P-value
College education rate	−0.0065	0.012	−0.031	0.018	0.595
ADI	3.30** × **10^ − 4^	1.21** × **10^ − 4^	9.31** × **10^ − 5^	5.67** × **10^ − 4^	0.006
White, %	0.041	0.010	0.021	0.061	<0.001
Median income, $	3.51** × **10^ − 7^	1.54** × **10^ − 7^	4.85** × **10^ − 8^	6.53** × **10^ − 7^	0.023
Privately insured, %	−0.033	0.015	−0.064	−0.003	0.031

A significant positive association was observed for ADI, where a 1-unit increase corresponded to a 0.00033 percentage point rise in utilization (β =  3.30 ×  10 ⁻ ⁴, p <  0.05). The percentage of White individuals also showed a significant positive association, with a 1% increase corresponding to a 0.041 percentage point rise in utilization (β =  0.041, p <  0.001). Median income demonstrated a small but significant positive association, with each $10,000 increase linked to a 3.51 ×  10^–7^ percentage point rise in utilization (β =  3.51 ×  10^–7^, p <  0.05).

In contrast, private insurance coverage and college education rate exhibited negative associations with utilization. A 1% increase in private insurance coverage corresponded to a 0.033 percentage point decrease in utilization (β =  -0.033, p =  0.031). The effect of college education rate was not statistically significant, with a 1% increase corresponding to a 0.0065 percentage point decrease (β =  -0.0065, p =  0.595).

## 4. Discussion

This study investigated the complex interplay between socioeconomic factors and pancreatic cancer patients’ access to specialized oncology services, examining patterns at both the community and hospital levels. We examined the influence of income, education, white population, private insurance coverage, and area deprivation on utilization patterns. Our findings corroborate previous studies that have identified socioeconomic status as a significant determinant of cancer care utilization and outcomes. Lower-income and lower-education populations have been shown to face disproportionate barriers to accessing high-quality cancer treatment, leading to poorer health outcomes. Moreover, studies have demonstrated that disparities in health insurance coverage contribute to unequal access to and utilization of cancer care services [27,28].

### 4.1. Community-level findings

Our findings demonstrate a positive association between income, education, and private insurance coverage with increased utilization of oncology services. Higher income likely facilitates access by enabling patients to afford out-of-pocket expenses. Similarly, higher educational attainment may correlate with improved health literacy and proactive healthcare engagement. This aligns with previous research showing that lower-income and lower-education populations face disproportionate barriers to accessing high-quality cancer treatment, leading to poorer health outcomes.

While income was positively associated with oncology service utilization in our linear regression analysis, in the analysis between income categories and utilization rate we observed the relationship to be non-linear. The patients with lower incomes did not have the lowest utilization rate. We saw a U-shaped curve, with those in the middle-income range having the lowest utilization. This suggests that the relationship between income and access to oncology services is complex and may be influenced by other factors beyond just financial resources. One possibility could be the availability of safety-net healthcare services like Medicaid [29,30] for the most disadvantaged individuals, which may help offset the barriers to access experienced by the poorest patients.

However, the percentage of White residents did not emerge as a significant independent predictor of utilization. This suggests that racial demographics may not exert a substantial influence by itself, especially once other socioeconomic factors are considered. Yet, if we take a step back and examine our system data, we see a different picture. Table 1 shows that White patients accounted for 83.17% of the total cohort. This heavy representation of White individuals indicates a lack of racial diversity, potentially restricting our ability to uncover or accurately assess racial disparities. Additionally, when examining the percentage of White residents at the zip code level, we observe a highly skewed distribution, with a mean of 90.6% (SD =  14.8%) and a median of 95.6%. The interquartile range further indicates that most zip codes have a percentage of White residents exceeding 90%, with a minimum of 1.9% and an upper quartile of 97.3%. This clustering of data in predominantly White zip codes underscores the homogeneity of the studied population and reflects regional demographic patterns.

Interestingly, when we turn to Fig 3, part D, we observe a slight but consistent negative correlation between the percentage of White residents and oncology service utilization. The regression line slopes downward, hinting that as the proportion of White residents increases, utilization decreases. However, the relationship is weak and fails to reach statistical significance. One possible explanation is that a dense concentration of data points exists near the higher end of the White rate (approaching 1.0), pointing to predominantly White regions. This heavy clustering might limit the variability within the dataset, making it difficult to detect more nuanced effects of race on service use.

Importantly, we observed a significant inverse relationship between ADI and utilization. Higher area deprivation, characterized by lower income and education levels, was associated with reduced oncology service use [20]. This underscores the critical need for targeted interventions to address the healthcare disparities experienced by individuals living in socioeconomically disadvantaged areas. Addressing the social and economic barriers that prevent access to specialized cancer care is crucial to ensure equitable access and utilization of these essential services, particularly for vulnerable populations residing in deprived neighborhoods.

The link between private insurance coverage and higher income levels further emphasizes the crucial role of financial resources in determining access to specialized oncology care. Individuals from higher income brackets, who are more likely to have private insurance, exhibited significantly greater utilization of these essential services. This finding highlights the potential for expanded insurance coverage to help mitigate the socioeconomic disparities observed in the utilization of specialized cancer care. Improving access to affordable and comprehensive health insurance, particularly for lower-income populations, could be an effective strategy to promote more equitable access to critical oncology services for all patients, regardless of socioeconomic background.

### 4.2. Hospital-level utilization patterns

In addition to examining community-level utilization trends, the hospital-level analysis provides insights into access to specialized oncology services and into the characteristics of individuals who developed pancreatic cancer and sought care within the studied health system. These findings highlight distinct patterns that reflect the interplay of socioeconomic, demographic, and healthcare-related factors.

One notable finding was the positive association between ADI and hospital utilization. A 1-unit increase in ADI was associated with a higher likelihood of seeking care within the hospital system. This relationship suggests that individuals from socioeconomically deprived areas may be at greater risk of developing pancreatic cancer, potentially due to the cumulative effects of environmental exposures, lifestyle factors, and limited access to preventive care. Once diagnosed, these individuals may also rely more heavily on centralized hospital-based care due to a lack of alternative healthcare resources in their communities. This dual role of ADI—indicating both increased disease burden and reliance on hospital care—underscores the structural inequities that shape health outcomes and access for vulnerable populations.

Conversely, private insurance coverage was negatively associated with hospital utilization, with a 1% increase in private insurance linked to a significant decrease in utilization. This finding may reflect differences in the healthcare pathways of privately insured individuals, who might have access to a wider array of healthcare providers and specialized services outside the studied hospital system [31]. Privately insured individuals may also have better access to early detection and preventive care, potentially reducing the severity or stage of disease at the time of diagnosis. These disparities highlight the influence of financial resources and insurance status not only on access to care but also on the likelihood of developing advanced pancreatic cancer requiring hospital treatment.

The role of racial demographics further underscores the complexity of hospital-level patterns. Unlike the weak negative association observed at the community level, the proportion of White residents exhibited a strong positive correlation with hospital utilization. This finding suggests that White populations may have higher representation among those developing pancreatic cancer and seeking care within the hospital system. This could reflect differences in underlying disease risk, health-seeking behavior, or systemic inequities that facilitate access for White populations while posing barriers for racial minorities. However, the heavy clustering of data points at the higher end of the White population rate (approaching 1.0) likely reflects the demographic composition of the hospital’s catchment area, limiting the variability needed to detect more nuanced racial effects.

Interestingly, median income emerged as a small but significant predictor of hospital utilization, with higher-income populations more likely to receive care within the hospital system. This association may point to the role of financial stability in navigating complex healthcare pathways and affording out-of-pocket expenses related to cancer treatment. However, the relatively modest effect size of income compared to ADI suggests that structural and neighborhood-level factors may exert a stronger influence on hospital utilization than individual financial resources alone.

Finally, the role of college education demonstrated a non-significant trend toward decreased utilization at the hospital level. This trend could reflect greater health literacy and proactive engagement with preventive care among highly educated populations, potentially lowering the risk of advanced pancreatic cancer requiring specialized treatment [19]. Alternatively, it may indicate that individuals from highly educated areas are more likely to seek care outside the studied hospital system, leveraging broader healthcare options.

**Fig 4:**
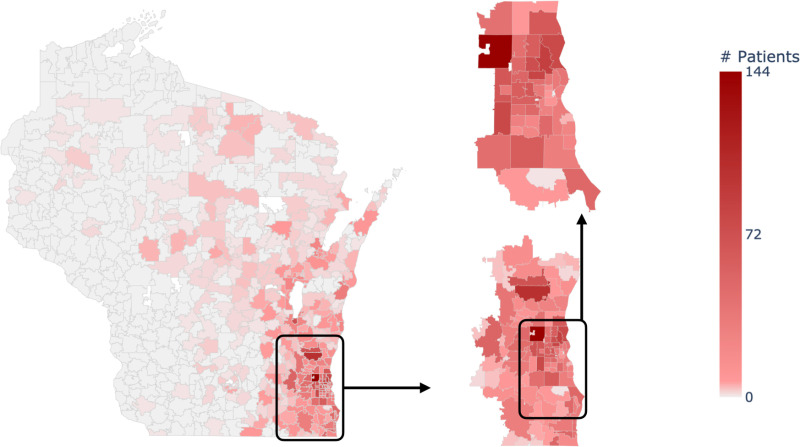
Pancreatic Cancer patients’ distribution across Wisconsin. The series of maps offers a detailed view of pancreatic cancer patient distribution in more focused areas of Wisconsin. The left panel serves as a reference for statewide population density. The right panels zoom in to display pancreatic cancer prevalence. Darker shades indicate higher patient counts in specific locales, providing insight into regional patterns and potential clusters within the state. These focused visualizations are critical for identifying areas with elevated incidences of pancreatic cancer.

Fig 4 displays the geographic distribution of pancreatic cancer cases across Wisconsin, juxtaposed against the backdrop of population density. The concentration of cases in the state’s southeastern region is immediately apparent, with a notably higher incidence in these areas compared to the rest of Wisconsin. The geographic distribution also reveals a pattern of disparity, with lower utilization observed in the more rural, sparsely populated regions. Regions with higher minority populations and greater socioeconomic deprivation appear to have reduced access to the specialized oncology services required for optimal management of this deadly disease.

### 4.3. Limitations

While our analyses were comprehensive, the findings require validation across diverse regions and healthcare systems. Our data was sourced from a single health system, reflecting the characteristics of a specific population, which may limit generalizability. To address this, we integrated U.S. Census Bureau data with Froedtert Health System data, incorporating zip code-level socioeconomic indicators such as median income, education rates, racial composition, and the Area Deprivation Index (ADI). This approach provided broader community context beyond the patient population. However, regional differences in socioeconomic and healthcare infrastructure may still influence these outcomes, necessitating further studies in more diverse settings.

### 4.4. Future work

To build upon this research, future studies should aim to incorporate data from multiple healthcare systems and regions to provide a more comprehensive understanding of the socioeconomic determinants of pancreatic cancer care utilization. Additionally, qualitative investigations, such as patient interviews and focus groups, could yield valuable insights into the lived experiences and barriers faced by individuals from various socioeconomic backgrounds in accessing specialized oncology services.

## 5. Conclusion

In conclusion, our study has revealed the complex interplay between socioeconomic factors and the utilization of specialized oncology services for pancreatic cancer patients. While income, education, and private insurance coverage were positively associated with higher utilization at the community level, area-level deprivation emerged as a significant barrier, underscoring the need for targeted interventions to address healthcare disparities in socioeconomically disadvantaged areas. Additionally, hospital-level findings highlighted the reliance of disadvantaged populations on centralized care systems and underscored disparities in care-seeking behaviors among racial and socioeconomic groups. Promoting equitable access to specialized cancer care—through strategies such as expanded insurance coverage, improved healthcare infrastructure, and community-based interventions—is essential to ensure optimal health outcomes for all pancreatic cancer patients, regardless of their socioeconomic background.
